# Correction: Applying ChatGPT to tackle the side effects of personal learning environments from learner and learning perspective: An interview of experts in higher education

**DOI:** 10.1371/journal.pone.0315764

**Published:** 2024-12-10

**Authors:** The PLOS ONE Staff

There are errors in the author affiliations. The publisher apologizes for these errors. The correct affiliations are as follows:

XiaoShu Xu^1^, XiBing Wang^2,3^, YunFeng Zhang^4^, Rong Zheng^3,5^

**1** School of Foreign Studies, Wenzhou University, Wenzhou, Zhejiang, China, **2** School of Information Engineering, Yunnan Vocational College of Mechanical and Electrical Technology, Kunming, Yunnan, China, **3** Graduate School, Stamford International University, Suan Luang, Bangkok, Thailand, **4** Centre for Portuguese Studies, Macao Polytechnique University, Se´ Freguesias, Macao, China, **5** School of Education, Baoshan University, Baoshan, Yunnan, China.
