# Supplementary material for: Applying ChatGPT to tackle the side effects of personal learning environments from learner and learning perspective: An interview of experts in higher education
Source: PLoS One. 2024 Jan 3;19(1):e0295646. doi: 10.1371/journal.pone.0295646 (PMC10763943; doi:10.1371/journal.pone.0295646)
Supplement: S1 Appendix — (DOCX) [file pone.0295646.s001.docx]

**Appendix Interview questions**

1. How do you understand Personal Learning Environments (PLEs) and ChatGPT?

2. In your experience, have you worked with PLE platforms before? If so, can you describe your experience with them?

3. How familiar are you with ChatGPT technology?

4. Do you think there is the possibility to incorporate ChatGPT technology into PLEs?

5. In your opinion, what are the benefits of incorporating ChatGPT technology into PLEs?

6. Have you experienced any challenges when implementing PLEs in higher education? If so, can you describe them?

7. What do you think are the challenges that all stakeholders, including students, instructors, and administrators, face when implementing PLEs in higher education?

8. How can ChatGPT be used to address the challenges learners face in keeping up with technological advancements and effectively using digital tools to find credible sources of information, particularly in relation to learners' digital literacy skills?

9. How can ChatGPT be used to develop non-cognitive skills such as motivation, perseverance, self-regulation, and self-efficacy in learners using PLEs?

10. How can ChatGPT be used to develop metacognitive skills such as self-determination, self-efficacy, and self-regulation in learners using PLEs, particularly in terms of their management of Web 2.0 tools and taking an active role in their learning outcomes?
