# Supplementary material for: Applying ChatGPT to tackle the side effects of personal learning environments from learner and learning perspective: An interview of experts in higher education
Source: PLoS One. 2024 Jan 3;19(1):e0295646. doi: 10.1371/journal.pone.0295646 (PMC10763943; doi:10.1371/journal.pone.0295646)
Supplement: Attachment — Submitted filename: Response to Reviewers.docx [file pone.0295646.s002.docx]

**Response to reviewers’ comments**

Dear Editor and Reviewer 1,

We greatly appreciate your thorough, insightful comments and constructive suggestions. We have modified the paper accordingly by addressing all comments and suggestions. Please see our detailed responses below.

Reviewer 1

Comments 1: Is the manuscript technically sound, and do the data support the conclusions? Yes.

Response 1: Thanks for your positive comment.

Comment 2: Has the statistical analysis been performed appropriately and rigorously? Yes.

Response 2: Thanks for your positive comments.

Comment 3: Have the authors made all data underlying the findings in their manuscript fully available? Yes.

Response 3: Thanks for your comments. We have fully provided the complete data in the manuscript, and all the interview transcripts have been sorted out and uploaded on the specified platform “figshare”.

Comments 4: Is the manuscript presented in an intelligible fashion and written in standard English? Yes.

Response 4: Thank you for your positive comment. We have invited a native English-speaking scholar to proofread the paper.

Comment 5: It is a current trend and good idea to explore the data. Data presentation could be made better by adding most relevant narrations of interviewee as examples. Discussion could be more supported by the previous research studies.

Response 5: Thanks for your suggestions. We have added more relevant narrations of interviewees as examples which were highlighted in yellow. For instance, to answer Question 6: Have you experienced any challenges when implementing PLEs in higher education? If so, can you describe them? We added:

*Professor Interviewee 2 further pointed out that:*

*“some students may lack consistent access to the necessary technology, including computers and reliable internet access. This digital divide can make it difficult for these students to fully participate in a PLE.”*

*Professor Interviewee 1 added:*

*“It's also important to continuously update the training as the technology evolves and as we gain more insights into effective teaching and learning strategies in digital environments. However, developing and providing this training requires a significant investment of time and resources, which can be a challenge in itself.”*

To answer Question 7: What do you think are the challenges that all stakeholders, including students, instructors, and administrators, face when implementing PLEs in higher education? We added professor interviewees’ opinions on the challenges administrators encountered:

*In terms of administrators, the professor interviewees mainly pointed out the following three challenges, including infrastructure and technical support, that is administrators need to ensure that the institution has the necessary technical infrastructure to support the implementation of PLEs. This includes reliable internet access and user-friendly platforms; second, professor interviewee 4 and 5 mentioned the challenge of resource allocation, which means when implementing PLEs, administrators need to allocate resources effectively to support a successful transition to PLEs; third, professor interviewee 3 and 6 highlighted the data privacy and security issue. professor interviewee 3 said:*

*“Protecting student data and ensuring privacy and security are crucial considerations when implementing PLEs. Administrators must address potential risks and ensure compliance with data protection regulations.”*

We also added the previous research studies to the discussion part, including research made by:

*Martins, J., Branco, F., Gonçalves, R., Au-Yong-Oliveira, M., Oliveira, T., Naranjo-Zolotov, M., & Cruz-Jesus, F. (2019). Assessing the success behind the use of education management information systems in higher education. Telematics and Informatics, 38, 182-193.*

*Prior, D. D., Mazanov, J., Meacheam, D., Heaslip, G., & Hanson, J. (2016). Attitude, digital literacy and self efficacy: Flow-on effects for online learning behavior. The Internet and Higher Education, 29, 91-97.*

*Akhyar, Y., Fitri, A., Zalisman, Z., Syarif, M. I., Niswah, N., Simbolon, P., ... & Abidin, Z. (2021). Contribution of Digital Literacy to Students' Science Learning Outcomes in Online Learning. International Journal of Elementary Education, 5(2), 284-290.*

*Stephen, J. S., Rockinson-Szapkiw, A. J., & Dubay, C. (2020). Persistence model of non-traditional online learners: Self-efficacy, self-regulation, and self-direction. American Journal of Distance Education, 34(4), 306-321.*

*Robinson, L. (2021). Investigating critical thinking disposition, self-efficacy, self-regulation, and self-identity amongst online students. College Student Journal, 55(3), 325-337.*
