# Supplementary material for: Applying ChatGPT to tackle the side effects of personal learning environments from learner and learning perspective: An interview of experts in higher education
Source: PLoS One. 2024 Jan 3;19(1):e0295646. doi: 10.1371/journal.pone.0295646 (PMC10763943; doi:10.1371/journal.pone.0295646)
Supplement: Attachment — Submitted filename: Response to reviewers-9.15.docx [file pone.0295646.s003.docx]

**Response to reviewers’ comments**

Dear Editor and Reviewers,

We greatly appreciate your thorough, insightful comments and constructive suggestions. We have modified the paper accordingly by addressing all comments and suggestions. Please see our detailed responses below.

**Reviewer 1:** Author did an effort to incorporate all the suggested changes in manuscript. This could be published by following format procedure of the Journal.

**Response:** Thanks for your positive comments and acceptance.

**Reviewer 2:** There are a few comments from this reviewer.

**Comment 1:** In the revised manuscript, lines 181 & 182, "To collect data, the researchers conducted online semi-structured interviews in 20 Febraury, 2023, using the Tencent Conference tool." It should say "...on 20 February, 2023,...". To correctly use the word 'on' and the spelling for 'February'.

**Response 1:** Thanks for pointing out the mistake. We have revised it as

“during 20-27 February, 2023”

**Comment 2:** Based on the first comment above, how was it possible to interview all nine participants in this study in one day? Are all nine available on that day of interview upon advanced arrangement or appointments for the interview date and different times? Was the interview done simultaneously by all the four authors/researchers and thus was done in a day? Please make the data collection clearer.

**Response 2:** Thanks for pointing out the carelessness. The interview was arranged during 20-27 February, 2023. We have revised it accordingly.

**Comment 3:** For 'Section 2.2 Research Instruments' - the terms 'open-ended questionnaire' (line 204), 'The questionnaire' (lines 205 & 210) and 'The survey questions' (line 219) are quite confusing for this qualitative study. The use of questionnaire would imply the respondents wrote or typed their responses in written form. However, it was stated that "The interviews were conducted in English and lasted between 25 to 30 minutes" (lines 183 & 184) and "The interviews were audio-recorded and transcribed for further analysis." (lines 186 & 187). And these subsequent statements informed this reviewer that actual face-to-face interactions or online interactions were conducted with all the nine respondents.

To avoid any further confusions to the readers, this reviewer suggests to remove all the terms referring to questionnaire and survey, and just say "The research instrument used for this study was an interview using ten open-ended interview questions (see Appendix 1). The questions aimed to gather data on the..."

Similarly, to rephrase the statements below accordingly:
"The interview questions was divided into three categories. The first set (Questions 1-3) aimed to understand the experts' definitions of PLE,..."

"The interview questions were reviewed by two experts in the field of Personal Learning Environments..."

**Response 3:** Thanks for your detailed recommendations. We have revised all the expressions on “questionnaire” and “survey” accordingly.

**Reviewer 3:**

**Comment 1:** I appreciate the work put into this new version of the article. However, I think comment 5 is not fully attended. For instance, questions 1-3 do not have any relevant narration included in the section where the responses to these questions are.

**Response 1:** Thanks for your explanation. We have added relevant narrations to questions 1-3, for instance, we added the following narrations to analyze question 1:

*For instance, expert interviewee 2 said:*

*“When you bring Chat into PLEs, it can interact with learners, answer their questions, and guide them through their learning. Like having a digital tutor 24/7”.*

*For instance, professor interviewee 3 pointed out that:*

*“They have the freedom to choose the digital resources and tools that best suit their learning styles and preferences. This customization empowers them to take ownership of their education and fosters a sense of responsibility for their learning outcomes.”*

And, we added the following narrations to analyze question 2:

*For example, in answering question 2, expert interviewee 3 mentioned that:*

*“I've seen how PLEs can help students take control of their learning, find resources and digital tools that fit their needs, and engage more deeply with the material. It's been very rewarding to see students become more active and self-directed in their learning.”*

*For instance, in answering question 2, professor interviewee 3 pointed out that:*

*“They have the freedom to choose the digital resources and tools that best suit their learning styles and preferences. This customization empowers them to take ownership of their education and fosters a sense of responsibility for their learning outcomes.”*

And, we added the following narrations to analyze question 3:

*For instance, expert interviewee 1 said:*

*“Making it engaging and user-friendly can truly amplify its effectiveness. You want to make sure that learners find it easy to interact with and feel comfortable asking questions. That's when the real learning happens.”*

**Comment 2:** The discussion section is still not fully supported. I mean there are assertions in the discussion that do not explicitly relate to the findings in the previous section. Is it possible to enhance this section? e.g. see lines 588-603 how the findings in the literature that you refer relate to your findings...There is not a clear link.

**Response 2:** Thanks for pointing out the mis-link. We have enhanced this part by referring to the literature review as well as adding new citations to explain our findings. For instance, we rewrote the first two paragraphs of the discussion part as:

*“Expert interviewee 2 suggests that ChatGPT can address these challenges by offering learners customized learning materials and instantly presenting relevant and credible information. Simultaneously, research emphasizes the value of proficient information management within PLEs [29]. As expert interviewee 3 notes, ChatGPT can fulfill this demand by supplying appropriate information to its users.*

*Moreover, prior research underscores the importance of digital literacy skills in online learning environments [30,31]. This mirrors challenges faced by learners in PLEs, who require substantial digital literacy to utilize the platform optimally. Expert interviewee 3 indicated that ChatGPT can recommend tutorials and additional materials to bolster learners' digital literacy, which resonates with findings from [19,25,26], suggesting that ChatGPT's tailored learning resources can enhance digital literacy.”*

As for Line 588-603, we rewrote as follows:

*“As for non-cognitive skills in applying PLEs, motivation, perseverance, self-regulation, and self-efficacy are proved to be crucial for learners to succeed in online learning [32,33]. Studies have emphasized the significance of these skills in online learning environment[21,34,35]. As the three professor interviewees pointed out ChatGPT, through personalized feedback and real-time assistance, could enhance learners' non-cognitive skills like motivation and self-efficacy in PLEs, aiding in goal-setting and overcoming challenges. Meanwhile, Nadira et al.[34] suggested that ChatGPT can offer motivational messages and tips to help learners stay motivated and persevere through challenging learning tasks[34].*

*In terms of metacognitive skills, setting individual learning objectives, resource management, and evaluating outcomes are regarded as crucial for success in PLEs [16]. This mirrors Kim's[36] observation that reflection allows students to be more attuned metacognitively to their cognitive activities, promotes profound comprehension, enables monitoring of acquired knowledge, values the learning journey, and assesses both the learning methodology and students' performance [36]. Besides, Erdogan[37] found that combining cooperative learning with reflective thinking activities can bolster students' critical and metacognitive skills [37]. Similarly, as pointed out by Educator interviewees 2 and 5, ChatGPT, by promoting a community feel via peer-to-peer learning, can aid in developing learners' socio-emotional capabilities such as empathy, clear communication, and collaborative teamwork. Moreover, ChatGPT can guide learners in pinpointing trustworthy information sources and assessing content quality, thus sharpening their critical thinking and making them judicious digital media consumers.”*

**Comment 3:** In the discussion, there is nothing about the possible role of tutors or academic support in helping learners build their PLE.

**Response 3:** Thanks for pointing out the role of teachers in PLEs. We added this in the discussion part as follows;

“*Concerning the roles that teachers or instructors assume in the evolution and application of PLEs in higher education, their primary transition should be from traditional "knowledge deliverers" to "learning facilitators/coaches". They must recognize the paradigm shift towards PLEs in higher education (Tavares et al.,2010) and equip themselves with foundational digital skills, the new educational philosophy (Haworth, 2016) (e.g., competence-based learning) and new pedagogical designs, updated Information and Communication Technology (ICT) (Yen et al., 2013), ensuring they can adeptly support learners in the development of PLEs.”*

**Comment 4:** No limitations of the study are found either.

**Response 4:** Thanks for pointing out the missing limitation part. We added as follows:

*“This study presents certain limitations: initially, the sample comprised only three ICT experts and six professors, possibly not encapsulating the viewpoints of other essential stakeholders in higher education like teachers and students. Additionally, the semi-structured interview format depended on participants' self-disclosures, potentially introducing biases and inaccuracies in their recollections and perceptions.”*

**Comment 5:** In line 425 I think there is a typo after "which means ...." Please check and reword if this is the case.

**Response 5:** Thanks for pointing out the confusion. We rewrote the part as:

*“…**…which means when implementing PLEs, administrators must strategically allocate resources to guarantee the effective integration of PLEs in higher education”*
