# Supplementary material for: Applying ChatGPT to tackle the side effects of personal learning environments from learner and learning perspective: An interview of experts in higher education
Source: PLoS One. 2024 Jan 3;19(1):e0295646. doi: 10.1371/journal.pone.0295646 (PMC10763943; doi:10.1371/journal.pone.0295646)
Supplement: Attachment — Submitted filename: Response to reviewers-10.4.docx [file pone.0295646.s004.docx]

**Response to reviewers’ comments**

Dear Editor and Reviewers,

We greatly appreciate your thorough, insightful comments and constructive suggestions. We have modified the paper accordingly by addressing all comments and suggestions. Please see our detailed responses below.

Reviewer #1: It is good to present all the possible outcomes of the study. However, heading 5 "conclusion and future research" only presents conclusion of the study. It is suggested to write clear future implications of the study or provide clear recommendations for future research.

Response: Thanks for your comment. We have added recommendations for future research in the last paragraph:

*“Regarding forthcoming studies, it's essential to recognize that ChatGPT is an emerging technology with its capabilities continually evolving. As a result, the general understanding of ChatGPT might not be exhaustive. Upcoming research might lean on experimental approaches and surveys to more deeply probe the extent to which ChatGPT can bolster areas like learners' digital literacy, non-cognitive facets, and metacognitive capabilities”.*

Reviewer #2: All my comments have been addressed as well as from other reviewers. Good improvement in this revised paper.

Response: Thanks for your positive comment.
